# Face mask uptake in the absence of mandates during the COVID-19 pandemic: a qualitative interview study with Swiss residents

**DOI:** 10.1186/s12889-021-12215-4

**Published:** 2021-11-26

**Authors:** Bettina Maria Zimmermann, Johanna Eichinger, Franziska Schönweitz, Alena Buyx

**Affiliations:** 1 University of Basel, Institute for Biomedical Ethics, Bernoullistrasse 28, 4056 Basel, Switzerland; 2 Technical University of Munich, School of Medicine, Institute of History and Ethics in Medicine, Ismaninger Str. 22, 81675 Munich, Germany

**Keywords:** COVID-19, Face masks, Public perception, Public health ethics, Public health policy, SARS-CoV-2, Solidarity, Switzerland

## Abstract

**Background:**

In the COVID-19 pandemic, Switzerland introduced broad nationwide face mask mandates only by October 2020, later than other Western European countries. This study aims to assess the underlying values and considerations of individuals to wear face masks in the absence of face mask mandates in the COVID-19 pandemic in German-speaking Switzerland.

**Methods:**

As part of the “Solidarity in times of a pandemic” (SolPan) research commons, we interviewed 31 participants living in the German-speaking part of Switzerland in April 2020 and 25 of them again in October 2020. Qualitative inductive thematic analysis was applied for data analysis and interpretation. Public health ethics principles guided the interpretation and organization of findings.

**Results:**

Five themes were identified: Trust and governmental policy; perceived benefits of mask-wearing; perceived risks of mask-wearing; social exclusion and prejudice; and decision-making in the absence of mandates. In light of increasing infection rates in October 2020, many participants started to consider the benefits higher than the risks and were willing to accept face mask mandates in that context, despite earlier reservations.

**Conclusions:**

The absence of face mask mandates underline individual autonomy but may also suppress personal responsibility due to social influence. Face masks are only temporarily acceptable in liberal Western societies and face mask mandates should be enforced only when epidemiologically necessary.

**Supplementary Information:**

The online version contains supplementary material available at 10.1186/s12889-021-12215-4.

## Key points

- People living in Western democracies need some time to integrate face masks as a protection against COVID-19 into their everyday lives.
- The absence of face mask mandates may make people consider the pros and cons of them more actively as authorities did not take these considerations off them.
- The absence of face mask mandates enhances individual autonomy but may suppress personal responsibility as people wearing them tend to feel socially isolated if only a minority wears them.
- Face masks contradict the Western understanding of social encounter and should only be enforced if epidemiologically necessary.

## Introduction

Face masks serve not only the purpose of preventing infections but also have social and cultural meanings [1]. In Europe, where, unlike in Asia, face masks were not commonly seen in public before COVID-19, face masks represent a visible sign of the ongoing pandemic and wearing or not wearing face masks demonstrate personal attitudes and compliance. As such, mask-wearing is commonly perceived as pro-social behaviour [2] and a solidaristic act of social cohesion [3, 4]. Nevertheless, Pfattheicher et al. (2020) [5] concluded from their quantitative inquiry on the role of empathy for mask-wearing in Western countries that it was difficult to measure to which degree mask-wearing was solidaristically or egoistically motivated. This mirrors a well-known divide in public health ethics: individualistic versus communal considerations [6]. Particularly in the context of epidemics and pandemics, public health scholars have called for relational, solidaristic views on public health ethics [7, 8], especially in the global health context [9].

At the beginning of the COVID-19 pandemic in Spring 2020, scientific evidence for the effectiveness on the general, broad use of face masks was lacking. Moreover, face mask stocks were scarce in many countries. Therefore, the World Health Organization initially did not recommend face masks for everyone [10]. By contrast, scientific experts early on recommended the general use of face masks [11]. Indeed, scientific evidence collected since the beginning of the COVID-19 pandemic supports the effectiveness of face masks for controlling the spread of the SARS-CoV-2 virus [3, 12, 13], particularly when used in combination with other containment measures [14].

The data presented in this paper was collected in German-speaking Switzerland in April and October 2020. Switzerland is a Western European country with nearly 8 million inhabitants. It is a direct democracy, meaning that people can directly vote and take referenda to decide legislation. In the absence of national regulations, the introduction of face mask mandates initially was in the responsibility of the 26 federal regions (cantons) and, consequently, varied from canton to canton. Due to the geographically small regions, regulatory differences between cantons were particularly noticeable as people are regularly travelling between regions. Switzerland represents an interesting case study as there were no broad national face mask mandates across the country until October 2020, later than other Western European countries (Table 1).

**Table 1:** Press releases from Swiss national authorities illustrate the temporal development of face mask policies during the COVID-19 pandemic until October 2020.

Date	Title of press release	Content summary
22 April 2020	Coronavirus: Federal Council does not want a general obligation to wear a mask	Swiss Federal council decides against face mask mandates
29 April 2020	Coronavirus: Federal Council to ease further measures from 11 May	Face masks start to be considered for certain branches when creating after-lockdown protection strategies
30 April 2020	New coronavirus: the ‘Protect yourself and others’ campaign moves to pink phase	Swiss health authorities recommend general face mask wearing for the first time, particularly in public situations when a distance of at least 2 meters cannot be respected
1 July 2020	Coronavirus: Masks compulsory on public transport; quarantine for travellers from high-risk regions; lifting of certain entry restrictions from 20 July	Nation-wide face mask mandates in public transport from 6 July 2020
18 October 2020	Coronavirus: Restrictions on private events, no gatherings in public of more than 15 people; masks mandatory in more areas and working from home recommended	Nation-wide face mask mandates in publicly accessible indoor areas
28 October 2020	Coronavirus: further measures to contain the epidemic, introduction of rapid testing, new rules on travel quarantine	Nation-wide face mask mandates extended to outside areas of establishments and facilities and busy pedestrian zones as well as schools (upper secondary level and higher)

This paper aims to qualitatively extract values and considerations inhabitants of German-speaking Switzerland applied to mask-wearing in the absence of face mask mandates in the COVID-19 pandemic. The following research questions are addressed: How did participants experience mask-wearing in April and October 2020? What values and evaluations did they assign to face masks and how did this change during the pandemic? How did they react to face mask policy and political communication? What practical implications do these findings have for public health policy, particularly face mask mandates, in Western democracies?

## Methods

### Recruitment and data collection

This study is part of the qualitative, longitudinal and multinational study “Solidarity in times of a pandemic” (SolPan) and has been made possible by the SolPan research commons. Participants were recruited through the websites of the universities and research groups participating in SolPan, snowballing and convenient sampling. Following a pragmatic approach [15], we addressed data saturation through analytical rigour and by controlling for a broad demographic representation when recruiting participants, aiming for a wide variety of perspectives (Table 2). Each participant was invited twice to give an interview in April and October 2020. All collaborating SolPan country teams used coordinated and collaboratively developed interview guides [16]. The interview guides included questions about changes in participants’ lives during the pandemic; their responses to COVID-specific rules and motivations to (not) follow them; observations concerning reactions of wider society; information behaviour and trustworthy sources; and future expectations. In October 2020, we specifically asked participants about COVID-19 vaccination, face masks as well as tracking and tracing. Interviewers were instructed to follow up on relevant topics not covered by the interview guide if applicable.

**Table 2:** Demographic distribution of interview participants

Demographic category	T1 (April 2020)	T2 (October 2020)
**Age**		
18–30	8 (25,8%)	5 (20%)
31–45	6 (19,4%)	5 (20%)
46–60	7 (22,6%)	7 (28%)
61–70	5 (16,1%)	4 (16%)
70+	5 (16,1%)	4 (16%)
**Gender**		
Female	16 (51,6%)	13 (52%)
Male	15 (48,4%)	12 (48%)
**Household**		
Single	8 (25,8%)	6 (24%)
Couple	10 (32,3%)	8 (32%)
Living with child (ren) < 12	3 (9,7%)	3 (12%)^a^
Living with child (ren) 12+	5 (16,1%)	5 (20%)
Other	5 (16,1%)	3 (12%)
**Rural/urban**		
Big town^b^	10 (32,3%)	8 (32%)
Medium/small town	6 (19,4%)	5 (20%)
Rural (e.g. village)	15 (48,4%)	12 (48%)
**Employment status**		
Employed with long-term contract	13 (41,9%)	11 (44%)
Self-employed	3 (9,7%)	3 (12%)
Employed with short-term/precarious contract	6 (19,4%)	4 (16%)
Unemployed	1 (3,2%)	1 (4%)
Retired	7 (22,6%)	6 (24%)
Other	1 (3,2%)	0 (0%)
**Education**		
Less than 10 years	10 (32,2%)	7 (28%)
10–14 years	3 (9,7%)	2 (8%)
Higher education	18 (58,1%)	16 (64%)
**Household net income**^**c**^		
Up to 4000CHF/month	6 (19,4%)	5 (20%)
4001-7000CHF/month	9 (29%)	7 (28%)
More than 7000CHF/month	16 (51,6%)	13 (52%)
**Total**	**31**	**25**^**d**^

Notes

^a^One participant gave birth to a child between T1 and T2, another participant living with young children dropped out for T2

^b^Defined as Swiss cities with more than 100′000 inhabitants

^c^Gross income minus social security contributions. Taxes are paid separately in Switzerland

^d^Six participants dropped out for T2 because they did not reply (*n* = 3), did not have time (*n* = 1), did not want to participate anymore (*n* = 1) or moved to another country (*n* = 1)

At least 2 days before the interview, all participants received a study information leaflet with in-depth information about the study. Before the interview, the interviewer answered any questions participants had concerning the study and consent was obtained orally. The audio of both the consent and the subsequent interview was recorded on a digital recorder and stored for transcription. Interviews lasted 30 to 45 min and were conducted by phone or video chat in standard German or Swiss-German. Interviews held in Swiss-German dialect were translated into standard German upon transcription. All transcripts were pseudonymized, no data was returned to participants.

### Interview coding and data analysis

Interviews were tagged in Atlas.ti 9.0 using a tagging scheme developed inductively by the SolPan research commons [17]. The tagging of each interview was checked by a second researcher for inter-coder consistency. In addition to manual coding, transcripts were searched for the terms “face masks” and relevant synonyms. Data analysed for this paper include those parts of the interviews about face masks as identified through the tagging scheme and the keyword search. The detailed analysis steps are presented in Table 3.

**Table 3:** Data analysis process

Step	Analytic step	Remarks
1.1	Manual interview tagging with SolPan coding scheme (Atlas.ti 9.0). Tagging was checked by a second researcher for consistency.	Coding scheme was inductively developed by the SolPan research commons [17] and consistently applied to all interviews
1.2	Automated interview tagging by key word search.	Key words used (in original language): Maske, Mund-Nasen-Schutz, Mund-Nasen-Bedeckung (mask, face nose protection, face nose covering)
2	Extraction of all quotations tagged with the code “FACE MASKS” from the tagging scheme	Export from Atlas.ti into a word document
3	Inductive development of preliminary research questions and data analysis framework (in excel) based on initial familiarization with data	First author (BZ); details presented in Supplementary file 1
4	Data analysis: structuring of data based on analytical framework, analytical memo	Second author (JE); data structuring in excel file, in parallel writing of analytical memo in word file
5	Inductive, descriptive presentation and organization of interview data	First author (BZ) based on JE’s work. See Supplementary file 1 for a more detailed overview
6	Iterative feedback among co-authors	Both written feedback and oral discussions
7	Mapping of descriptive themes with public health ethics framework	Abductive; inductive themes were mapped with existing public health framework. Preliminary research questions were adapted to give the analysis are more narrow and relevant focus. See Supplementary file 1 for more details
8	Iterative feedback among co-authors	Both written feedback and oral discussions

These data excerpts were initially analysed following an inductive thematic analysis approach [18]. In the later analysis steps, (step 7 in Table 3) established public health ethics principles were used to organize and interpret inductively derived findings. The four *Principles of Biomedical Ethics* [19] have been commonly applied in the context of public health ethics. They include (1) *beneficence* (maximize individual and societal benefits); (2) *nonmaleficence* (avoid harm, if necessary weigh risks against benefits); (3) *justice* (fair and equitable distribution of health care); and (4) *respect for autonomy of the person* (respect an individual’s self-determination to make voluntary decisions based on understanding without undue external influences). Because these four principles were designed for the context of research and clinical ethics and have been criticized to be insufficient for the public health context [20], we additionally considered (5) the *principle of solidarity* [21] and (6) the *precautionary principle*, which aims to prevent harm by anticipating and controlling risks and by taking responsibility for future consequences of present actions [22].

To overcome individual bias and gain inter-personal reliability (understood as “consistency within the employed analytical procedures” for qualitative research [23]), data analysis was characterised by iterative circles of feedback among the first author and the co-authors, constant memo writing and critical reassessment of preliminary findings. Participants were not proactively asked for feedback during the analysis process because analyses on other topics have been conducted on the same material and we did not consider it appropriate to constantly re-contact them.

## Results

We qualitatively extrapolated five themes from the interviews that represent underlying values and considerations of individuals to wear face masks in Switzerland in April and October 2020: Trust and governmental policy; Perceived benefits; Perceived risks; Social exclusion and prejudice; and Decision-making in the absence of mandates (see Table 4 for an overview of findings).

**Table 4:** Overview of findings

Theme	April 2020	October 2020
Trust and governmental policy	Uncertainty due to contradicting communication from health authorities and scientific experts regarding effectiveness of face masks	Diminished trust in health authorities Contradicting communication and absence of mandates caused continued uncertainty concerning usefulness of face masks
Perceived benefits	Uncertainty about benefits	Protecting self and others; reducing risk of infection; reminder of pandemic; being able to go out
Perceived risks	Hardly considered	Concerns about face masks becoming "normal"; anonymity; wrong handling
Social exclusion and prejudice	Participants ridiculed mask-wearing people for being hysterical	Absence of mandates led to low uptake ➔ socially awkward to wear a mask
Decision-making in the absence of mandates	Hardly any decisions taken (lack of information and evidence)	Absence of mandates made participants make individual decisions when to wear them

### Trust and governmental policy

The way the Swiss Federal government had been handling face mask policies led to impaired trust that affected both the Federal government but also the usefulness of face masks in general. This issue was represented in different forms in April and October 2020.

In April 2020, several participants criticized the government for communicating at the beginning of the pandemic that masks were useless. They perceived this as an excuse as there were not enough masks available at the time.

[In Switzerland], information is not passed on correctly. For example, concerning the face mask. They just say that masks were unnecessary because we simply don’t have enough masks for everybody. And that [communication] annoys me. (P19_T1)

As a result of contradicting recommendations from health authorities and health experts concerning face masks, several participants expressed uncertainty about the usefulness of face masks in April 2020: “You don’t know, does it help, or does it not?“ (P11_T1).

In October 2020, many participants were critical of the absence of national face mask mandates, stating that inconsistent regulations were confusing and diminished trust in authorities further: “There is this feeling that a common thread or a strong hand that coordinates everything is missing” (P11_T2). One participant described their experience living at the border of two cantons with differing regulations:

So the regulations are cantonal and they differ quite a lot. You just have to consider that [Canton 2] borders [Canton 1] and [in Canton 1] there have been no face mask mandates until now. And it's quite strange […] On the other side [of the cantonal border] it's completely different. That is, you see even fewer masks […]. And that is problematic in my opinion because these inconsistencies mean that people are less accepting of the whole issue. (P23_T2)

Some participants remained uncertain about the usefulness of face masks even in October 2020, stating that they did not “know anymore what to believe concerning mask-wearing” (P21_T2) or that they were “sceptical if face masks really make sense but probably they do, ultimately”, (P24_T2). Some also doubted the usefulness of face masks due to the absence of national mandates or were observing that neighbouring countries with stricter face mask policies were not performing better concerning daily infection rates:

There are countries, even in Europe, that have had face mask mandates for much longer. For instance, Germany. Nevertheless, the numbers rise, sometimes exponentially, even in Germany. That would be like striking proof that the effectiveness is really not that great, or that people don't wear the masks properly. (P14_T2)

Consequently, communication from health authorities inconsistent with scientific evidence paired with a political hesitancy to enforce national face mask mandates led to a lasting uncertainty regarding the usefulness of face masks. This influenced some participants’ risk-benefit assessment concerning their face mask use.

### Perceived benefits

In contrast to the doubts expressed by some participants, others were “convinced that it’s going to make a difference”, (P19_T2). Several participants stated that they wore face masks not only to protect themselves but also for others, particularly at-risk individuals. This indicates both an individual as well as a solidaristic motivation for face mask-wearing. Even though perceived as exhausting and inconvenient at times, some saw mask-wearing as a moral obligation, as something “we should just do now” (P02_T2). „I think that’s part of everybody’s solidarity; everybody just has to do that [wear masks] now. To get the numbers down” (P20_T2).

While some participants perceived face masks as a source of certainty to prevent infection and said they were feeling safer when wearing one, others stressed that masks only reduced the risk for infection but could not prevent it. For instance, one participant stated that wearing a face mask alone was not a panacea, but in combination with other measures using them meant being “on the safe side” (P18_T2). Some participants expressed uneasiness when going into shops where very few people wore a face mask. Accordingly, several participants interviewed at the end of October 2020 expressed relief once general face mask mandates were in place: “I was just happy when the general mask mandates were introduced”, (P19_T2). These individuals assigned a high individual health benefit to face masks and were interested in a broader uptake, which they perceived as possible only if face mask mandates were enforced.

Some participants wore face masks to maintain some freedom, for instance, to still go out to meet people, go to the theatre or on vacation despite the pandemic. Moreover, several participants stated that face masks, if anything, served as a reminder for people about the ongoing pandemic to remain careful: “Well, I think, say three-quarters real protection… but otherwise very much symbolic, psychological. To just know we can’t act the way we normally do” (P21_T2). This indicates that benefits were not only limited to infection protection, which in some cases relativized the doubts remaining from inconsistent governmental communication (see theme 1) but include an additional symbolic dimension.

### Perceived risks

By October 2020, none of the participants felt strongly inhibited by face masks, refused to wear them or were strongly against face mask mandates even though some of them said that it was “inconvenient” or “strange” to wear a face mask. Some participants who were wearing face masks regularly for professional reasons or had stayed in a country with strict face mask mandates stated that they were getting used to it pretty fast and started to wear them in Switzerland as well.

However, participants expressed concerns about the side effects of face masks: First, some were concerned that face masks might become "normal" in the future, which they would find “horrible” (P02_T2), as it would constantly signal danger. Of note, several participants referred to Asia, stating that they would not want to live in a society where face masks were ubiquitous: “I hope that at least 60 or 70% of people get vaccinated so that we don’t have a culture to constantly wear a mask like in Asia” (P21_T2).

Anonymity was a second side effect of face masks mentioned by many participants. “Yes, that’s what happens when you wear a mask so often, you no longer see people’s facial expressions or their whole faces. That’s what’s missing. That does something” (P20_T2). Relatedly, several participants were concerned about the effects face masks might have on children or patients with dementia and psychiatric illnesses. “The residents [of nursing homes] have a hard time [...] when they only see their relatives while wearing masks. That also causes a lot of psychological problems” (P01_T2).

Third, some participants expressed concerns about the improper use of face masks. Seeing others wearing or handling face masks the wrong way was perceived as counterproductive. “If you wear a textile mask all the time and don’t wash it, it ends up carrying the bacteria around and then you put it down somewhere and then you have the bacteria there... I don’t know if that’s helpful” (P24_T2).

Despite these perceived side effects, most participants were generally in favour of implementing national face mask mandates, considering the potential benefits to be higher than the risks. Yet, feelings of social exposure were a hindering factor to mask-wearing.

### Social exclusion and prejudice

Many participants observed that in the absence of face mask mandates, few people wore a mask voluntarily. Several participants described how they were feeling strange when they were the only ones around wearing a mask. Some were feeling stigmatized as they feared others would think they were sick or infectious: “I think that people look at you rather askance if you wear a mask. You are considered infectious if you wear one” (P13_T2). For some, this was a considerable limitation to wearing a mask. Several participants were confident that once face masks were compulsory, everybody would start wearing them, removing this hindering factor of social exclusion: “I claim that many people would wear a mask if it was compulsory. And because it isn’t, they make a fuss and say, ‘what are people going to say if I walk around with a mask?’”, (P11_T2).

In some cases, participants were stereotyping others for not complying with face mask regulations. In October 2020, some participants generally accused “the young people” (P24_T2) or “the elderly” (P19_T2) of being particularly reluctant of wearing face masks. Observing others not or improperly wearing face masks was immediately seen as asocial and egoistic behaviour and interpreted as a sign of resistance:

One day, we took the train and there were four other people in our compartment. […] They didn’t wear a mask and I gave them a bit of an angry glance, but I didn’t say anything. And they immediately said ‘no, we don’t wear masks, it’s no use anyway’. And I was just aghast, I thought that’s just not… It’s like these [anti-COVID-19] demonstrations. There are such people. But it’s not just to protect yourself, but also to protect others. That’s a real concern for me. (P15_T2)

In April 2020, by contrast, most participants did not wear face masks at all. Some even ridiculed other people who, in their opinion, wore face masks to an exaggerated extent, for example in the forest or alone in the car. To them, this was a sign of excessive protection or even "hysterical" behaviour: “When I see people driving with a mask, I think that’s a bit over the top. I don’t see the point in wearing a mask when driving alone in a private care”, (P14_T1).

Therefore, the perceived socially desirable behaviour drastically changed from April to October 2020: while in April 2020, people sometimes criticised others for wearing a mask, in October 2020 it was the other way around.

### Decision-making in the absence of mandates

Due to the largely unregulated policy landscape concerning face masks, many participants described how they decided on when to wear a face mask in October 2020. While few participants stated that they were wearing face masks only “because one has to” (P22_T2), others spontaneously considered situations when they considered it necessary to wear one:

[…] it's completely spontaneous according to my instincts. If I have a feeling that I need to keep my distance or put on the mask, then I do that, of course. That’s just a momentary decision. (P15_T2)

Several participants stated that they were considering wearing a mask more often due to increasing infection rates in October 2020. This indicates that perceptions of infection risks were guiding their decision to wear a face mask. Following governmental recommendations, many participants stated that they were wearing a face mask if distances could not be kept, in crowded places, or when having symptoms of a cold. Several mentioned that these recommendations were making sense to them and appreciated the freedom to assess themselves when a place was crowded enough to wear a mask. One participant even said that they would be more motivated to wear face masks voluntarily because they felt that existing regulations were well-balanced.

In summary, the main reasons people mentioned for wearing face masks were to protect themselves and others, to prevent harm, for the common good, and because they could make their own choices when to wear them. They were reluctant to mask-wearing if they doubted their effectiveness or when fearing stigma and social isolation. Several participants stated that they would not wish to permanently see face masks in public places but were fine with it temporarily.

## Discussion

The findings show that the absence of face mask mandates triggers a sense of personal responsibility but also potentially leads to perceptions of social exclusion. Our findings also illuminate the role of trust in authorities and governmental communication, suggesting that diminished trust paired with the absence of face mask mandates in the Swiss context led people to self-examine the usefulness of face masks even beyond medical effectiveness.

Despite increasing scientific evidence about the airborne transmission of SARS-CoV-2 [24] and steadily increasing daily infection rates in summer and autumn 2020 in Switzerland [25], interview participants demonstrated an intensive individual cost-utility assessment when considering when to wear a mask in October 2020. Participants reported extensively on perceived risks and benefits of mask-wearing on both an individual and a societal level. Instead of mandates, Swiss authorities such as the Swiss Health Minister called for personal responsibility: “No communal spirit without personal responsibility” [26]. For some participants, this autonomy served as a motivator to adhere to existing rules and recommendations in general [27].

Yet, despite the repeatedly stressed importance of personal responsibility and autonomy in Swiss COVID-19 policy-making, many participants were glad when extensive face mask mandates came into force. The absence of face mask mandates further solidified existing uncertainties about the usefulness and medical effectiveness of face masks as a measure to protect against viral infection and led to social barriers hindering people generally willing to wear face masks to consistently wear them. These social barriers worked against the idea to strengthen individual autonomy in the form of personal responsibility. According to the *theory of social influences*, well-established in social psychology, people commonly rely on others from an informational and normative perspective to adjust their behaviour in public [28]. Consequently, the absence of face mask mandates tended to make those wearing face masks feel socially excluded. Similar tendencies were found in Germany before face mask mandates were introduced [2] and other studies confirm the communal and cultural effects on face mask uptake [29–32].

The contradicting messages sent by Swiss health authorities caused a loss of trust in their competence concerning face masks. This was also reflected in national population surveys, reporting a drop in respondents who trust the government from 70% in Spring to 36% in October 2020 [33]. The Swiss context contrasts existing evidence suggesting that overall trust in scientists and the government were critical factors for complying with face mask usage [1, 34, 35]: participants indicating diminished trust in authorities and persisting uncertainties concerning the effectiveness of face masks were still willing to wear masks and even favoured nation-wide mandates, as the majority of citizens did in October 2020 [33]. According to a longitudinal national survey, while trust in authorities decreased, acceptance of face mask mandates – for instance in stores – increased from 32% in June to 72% in October 2020 [33]. A plausible explanation for this observation is that people proactively identified other motivational factors to wear face masks that balanced out uncertainties about their epidemiological effectiveness, including precautionary considerations [22], a psychological feeling of protection [36] or a general reminder of the pandemic [37]. Moreover, COVID-19 related research on policy compliance also finds that shared social norms and values [38] as well as a sense of togetherness [27] support compliance with protective measures such as mask-wearing [39]. Similarly, Betsch et al. [2] found that mask-wearing was perceived as prosocial behaviour in Germany. Our findings demonstrate that people also use moral reasoning beyond cost-utility assessments by stressing that face masks symbolize social togetherness and mutual support, thereby referring to existing models of solidarity [21].

### Strengths and limitations

This is a qualitative study that seeks to understand a phenomenon in-depth rather than to aim for statistical representativeness. The qualitative nature of this study allows the assessment of this phenomenon from a socio-ethical perspective and maps the complexity of the phenomenon. Inductive findings are embedded in the theoretical context of public health ethics, allowing for a more practical and nuanced understanding of public health policy.

Switzerland represents a Western liberal democracy for examining people’s perceptions of face masks in the absence of face mask mandates. As such, findings may be transferrable to other Western democracies, but the cultural, political, economic and epidemiological contexts are important to consider as well. Of note, we interviewed people living in the German-speaking part of Switzerland but not from the other Swiss language regions. This is a limitation to the scope of the findings, as culture and political attitudes are known to differ between Swiss language regions [40]. Therefore, findings might have been different if we had interviewed people living in the French or Italian-speaking regions of Switzerland.

Our sample is also skewed towards the “better off”: people with high education, income and permanent employment contracts are overrepresented, whereas the unemployed, people working in precarious jobs or having small children are underrepresented. Those participants were particularly difficult to recruit as several potential participants had cancelled or declined participation due to a lack of time or energy. Moreover, all participants were at least to some extent willing to adhere to measures, and none of them identified themselves as a ‘COVID denier’. Yet, we did recruit participants from all socioeconomic groups (Table 2) and addressed the overrepresentation of the “better off” by purposefully screening data for alternative views during data analysis.

## Conclusion

The absence of face mask mandates strengthened individual autonomy (as suggested in theme 5 in the results section) but also suppressed personal responsibility as people tended to be nudged against wearing masks rather than wearing them in some circumstances (theme 4). Observing and feeling this tendency triggered participants to prefer mandates over individual autonomy, even in German-speaking Switzerland where personal responsibility is a deeply encultured value. Importantly, it might be this personal responsibility that maintained people’s willingness to comply despite lack of trust in government concerning face masks (theme 1): The absence of mandates made participants consider the pros and cons more actively (themes 2 and 3) as authorities did not take these considerations from them. In the long term, this might have positive effects on compliance, as long as people perceive the individual and communal benefits of existing regulations and mandates as proportionate to their costs. Yet, in the specific case of face masks, people living in Western democracies need some time to integrate them into their everyday lives. Face mask mandates may accelerate this habituation period.

Still, policymakers should be aware that face masks might contradict the Western understanding of social encounters. Our study indicates that face masks were perceived as a symbol of social disruption incoherent with Swiss culture. For instance, the notion that social exchange was impaired with face masks as facial expressions were more challenging to read was perceived as an important cost. Consequently, face masks were perceived acceptable only temporarily and it seems to be important that face mask mandates are only enforced when epidemiologically necessary in countries where they are not already culturally embedded. Hence, face mask mandates are an important instrument for viral containment but our findings indicate that they might be best enforced only as long as necessary in countries with Western culture.

## Supplementary Information

**Additional file 1.**

## Data Availability

The datasets generated and analysed during the current study are not publicly available as whole interview transcripts (raw data) cannot be completely de-identified. Legal restrictions apply to protect the identity of the participants as their consent was bound to confidentiality.
