# Supplementary material for: Face mask uptake in the absence of mandates during the COVID-19 pandemic: a qualitative interview study with Swiss residents
Source: BMC Public Health. 2021 Nov 26;21:2171. doi: 10.1186/s12889-021-12215-4 (PMC8620306; doi:10.1186/s12889-021-12215-4)
Supplement: Supplementary file 1 — Additional file 1. [file 12889_2021_12215_MOESM1_ESM.docx]

# Supplementary methods

This document supplements the methods section, particularly Table 3, of the paper “Face mask uptake in the absence of mandates during the COVID-19 pandemic: a qualitative interview study with Swiss residents”.

# Step 3: Preliminary research questions

- What general attitudes did participants express towards face masks?
- What moral norms and values did participants attach to face masks?
- What references or sources were participants referring to concerning the usefulness and wearing of face masks?
- What attributes did participants ascribe to face masks? How did they differ between regions with face mask obligations and those without?
- What were people´s reflections on the voluntary vs mandatory use of face masks?
- How did the perception, use and attitudes towards face masks change in German-speaking Switzerland between April 2020 (lockdown related to first pandemic wave) and October 2020 (beginning of second pandemic wave)?

# Steps 3-4: Preliminary analytical framework

- Summary of attitudes and views towards face masks (T1)
- Summary of attitudes and views towards face masks (T2)
- Notes on longitudinal changes in views
- Expressions of moral values/norms in relation to face masks
- References or sources participants referred to concerning the usefulness and wearing of face masks (e.g. Health authorities, media, studies, experts, friends...)
- Attributes participants ascribed to face masks (e.g. protecting themselves / protecting others / visible sign of compliance or noncompliance)
- General attitudes participants expressed towards face masks (wear them even voluntarily, only if obligatory, only reluctantly, not at all, etc.)
- Other relevant aspects

# Step 5: descriptive presentation of interview data

The here-presented figures are graphical overviews. Analytical memos, reconsiderations of primary data and group discussions accompanied each analytic step.

### Version 5.1


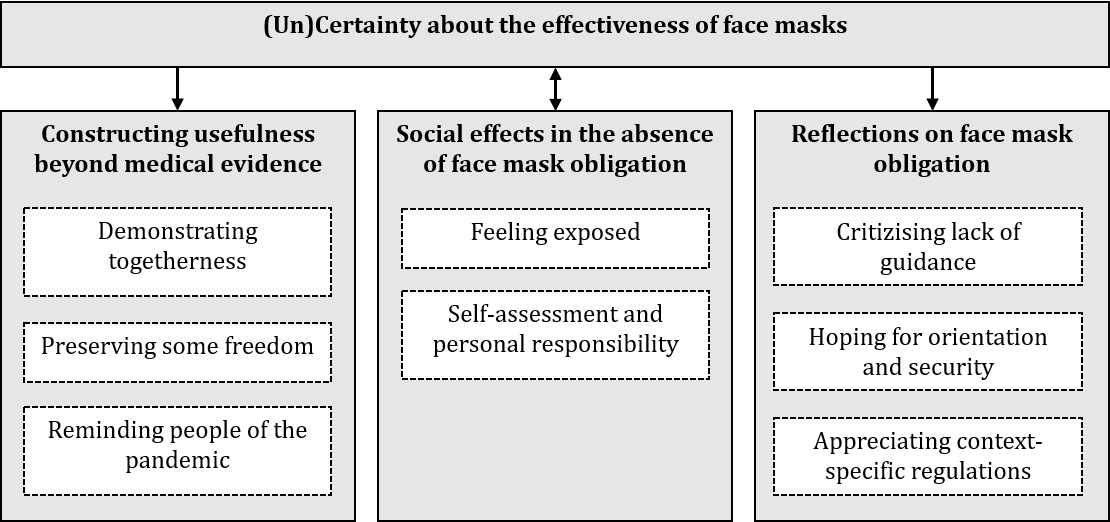


### Version 5.2


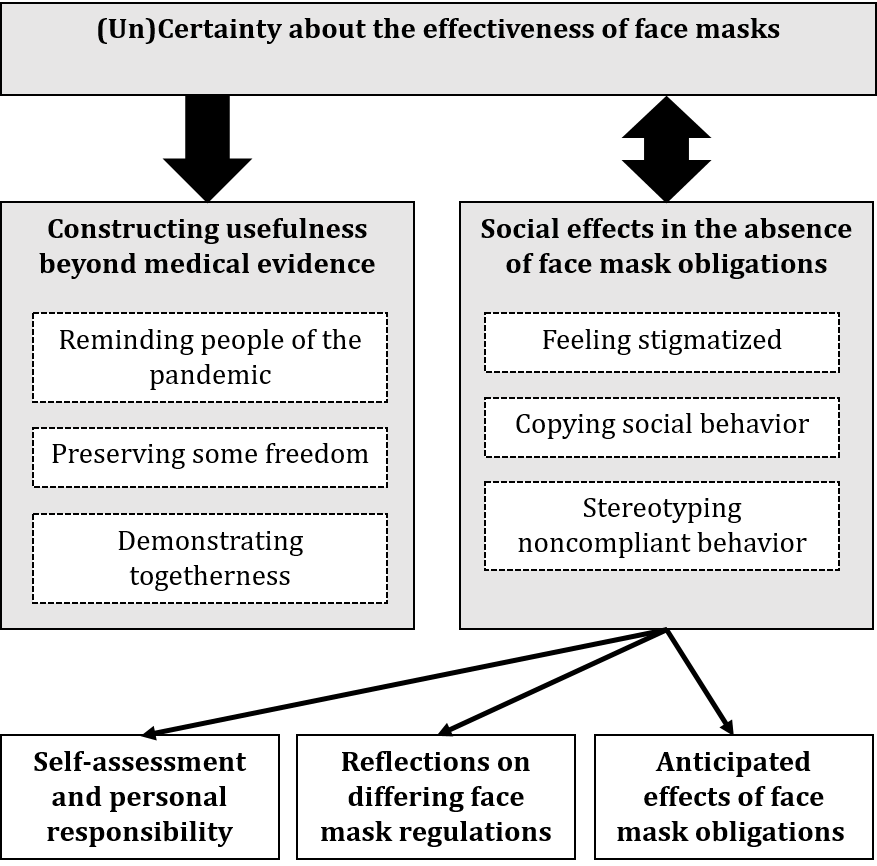


*Version 5.3*

*:*


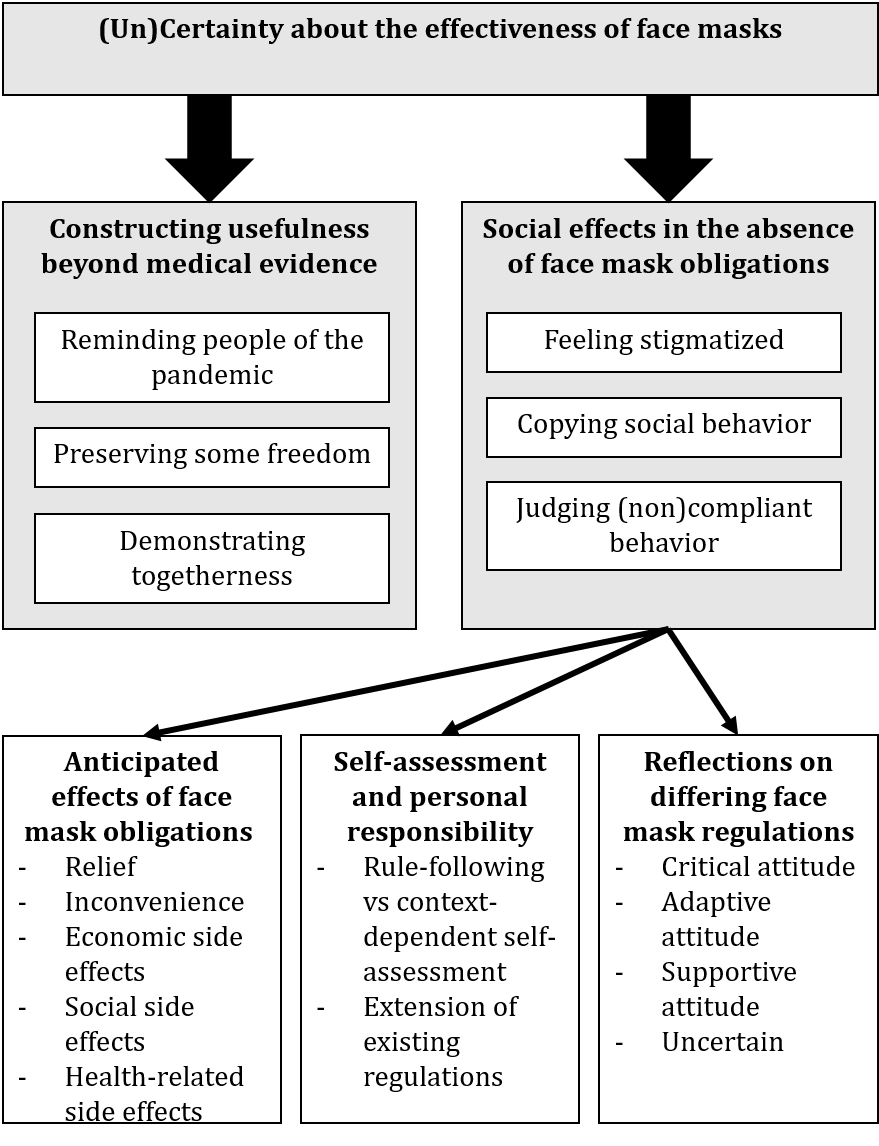


### Version 5.4


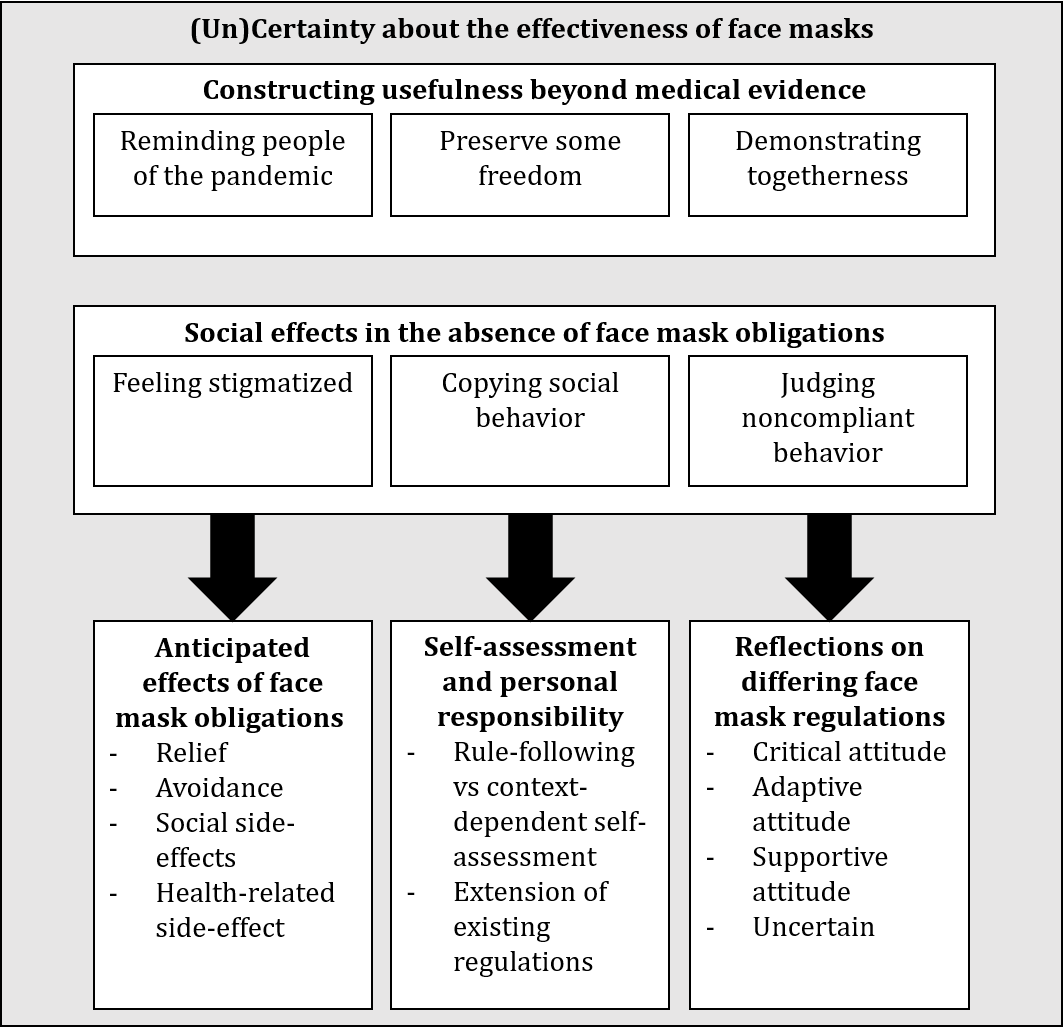


## Step 7: mapping with public health ethics framework

Adapted research question: *What values and considerations motivate or hinder people to wear face masks if they were not mandated by policymakers?*

| **Descriptive theme from interview data (step 1)** | **Mapping with public health ethics framework** | **Presented theme** |
| --- | --- | --- |
| (Un)Certainty about the effectiveness of face masks | Trust and transparency | Trust and governmental policy |
| (Un)Certainty about the effectiveness of face masks  Constructing usefulness beyond medical evidence | Beneficence | Perceived benefits |
| (Un)Certainty about the effectiveness of face masks  Anticipated effects of face mask obligations | Nonmaleficence | Perceived risks |
| Social effects in the absence of face mask obligations  (Un)Certainty about the effectiveness of face masks | Justice | Social exclusion and prejudice |
| Self-assessment and personal responsibility  Reflections on differing face mask regulations  Anticipated effects of face mask obligations | Autonomy and personal responsibility | Decision-making in the absence of mandates |
